# Dynamically Reconfigurable Data Readout of Pixel Detectors for Automatic Synchronization with Data Acquisition Systems

**DOI:** 10.3390/s20092560

**Published:** 2020-04-30

**Authors:** Farah Fahim, Simone Bianconi, Jacob Rabinowitz, Siddhartha Joshi, Hooman Mohseni

**Affiliations:** 1 ASIC Development Group, Particle Physics Division, Fermi National Accelerator Laboratory, Batavia, IL 60510, USA; 2 Bio-Inspired Sensors and Optoelectronics Laboratory, Northwestern University, 2145 Sheridan Rd, Evanston, IL 60208, USA; simone.bianconi@u.northwestern.edu (S.B.); JacobRabinowitz2021@u.northwestern.edu (J.R.); hmohseni@northwestern.edu (H.M.); 3 Department of Electrical and Computer Engineering, Northwestern University, 2145 Sheridan Rd, Evanston, IL 60208, USA; sidjos@u.northwestern.edu

**Keywords:** imaging, reconfigurability, detectors, photon counting, synchronization, adaptive-autonomous control

## Abstract

Reconfigurable detectors with dynamically selectable sensing and readout modes are highly desirable for implementing edge computing as well as enabling advanced imaging techniques such as foveation. The concept of a camera system capable of simultaneous passive imaging and dynamic ranging in different regions of the detector is presented. Such an adaptive-autonomous detector with both spatial and temporal control requires programmable window of exposure (time frames), ability to switch between readout modes such as full-frame imaging and zero-suppressed data, modification of the number of pixel data bits and independent programmability for distinct detector regions. In this work, a method is presented for seamlessly changing time frames and readout modes without data corruption while still ensuring that the data acquisition system (DAQ) does not need to stop and resynchronize at each change of setting, thus avoiding significant dead time. Data throughput is maximized by using a minimum unique data format, rather than lengthy frame headers, to differentiate between consecutive frames. A data control and transmitter (DCT) synchronizes data transfer from the pixel to the periphery, reconfigures the data to transmit it serially off-chip, while providing optimized decision support based on a DAQ definable mode. Measurements on a test structure demonstrate that the DCT can operate at 1 GHz in a 65 nm LP CMOS process.

## 1. Introduction

In large detector systems, information normally flows in one direction. Incoming particles or photons interact with the sensor material to generate charge, which is processed by a readout integrated circuit (ROIC) and subsequently transmitted to a data acquisition (DAQ) system for storage or further processing [1]. These detectors and DAQ systems often operate under varying environmental conditions, workloads, and with a variety of sensors. A static system cannot be expected to be power and bandwidth-efficient under all conditions [2] and hence reconfigurability of the ROIC and sensor is highly desirable [3,4,5].

Moreover, detector systems used in high energy physics (HEP), photon science, infrared imaging, internet of things (IoT) devices, etc., typically generate very large amount of data, on the order of Tbps [6,7]. This has increasingly driven the need for edge computing architectures that process the data at the source and reduce the amount of data that must be sent across long networks to data centers or clouds [8]. Efficiency is especially critical in IoT applications, where both energy and bandwidth are extremely limited resources [9,10,11].

Increasingly complex science applications are exponentially raising demands from underlying detector systems for optimized dataflows. To enable real-time performance (tens to hundreds of nanosecond time scales) detectors need to be faster, efficient, and more proactive, responding to bottlenecks before they become significant. Therefore, to enable optimized data collection and dynamic data reduction at source, adaptive feedback and control of the sensor and ROIC by the DAQ must exist. For example, in imaging detectors, if the number of incoming photons drops significantly, the detector could be triggered to change from full frame imaging to zero-suppressed readout. Alternatively, the window of exposure (time frame) could be increased to integrate over a longer duration, or the number of bits of information sent off chip could be reduced. These procedures would save both bandwidth and power by reducing the amount of data that is not of interest for transmission off-chip. ROICs with reconfigurable readout must be able to transition between readout modes or to adjust time frames without corrupting the data currently being read out. It is also crucial for the DAQ system to be synchronized without requiring large overhead, such as lengthy headers, whenever the detector readout is reconfigured. Additionally, the DAQ system should be able to automatically discern the start of a new readout frame while maintaining lock with the incoming data stream. In summary, there is a clear need for dynamically reconfigurable ROICs with auto-synchronization capabilities. In imaging systems, reconfigurable ROICs that can not only optimize data bandwidth but also reconfigure sensors will benefit a wide range of applications. For instance, detector systems with sensors capable of operating either in a passive imaging mode or in a dynamic ranging mode would enable foveation [12], resulting in a new class of high-resolution, high-frame rate, data-efficient, self-learning detection systems.

In this paper we present a technique for seamlessly changing dynamic time frames, readout modes and data packet length of a ROIC without data loss or corruption, and without requiring the DAQ to stop and resynchronize. Data throughput is maximized by using a minimum unique data format to differentiate between consecutive frames, rather than requiring lengthy frame headers. To test reconfigurability and auto synchronization with the DAQ system, a test structure of the data control and transmission (DCT) block has been implemented in a 65 nm LP CMOS process.

The rest of the paper is arranged as follows: in Section 2 we briefly discuss programmability in existing systems and their limitations. Section 3 describes the concept of a reconfigurable detection system enabled by Electron Injection (EI) sensors and programmable readout modes of the ROIC. In Section 4, we present the data control and transmitter (DCT) and the implementation of reconfigurability in the ROIC and auto synchronization with the DAQ. Conclusions follow in Section 5.

## 2. Programmability in Existing Systems and Its Limitations

Feedback mechanisms to achieve data reduction at the source are extensively applied in HEP experiments. In the Compact Muon Solenoid (CMS) detector, this is achieved by using a trigger mechanism based on the detected energies to select the potential physics of interest, from GHz interaction rate of collisions down to 100 kHz [13]. The ROIC stores information in local memory and waits for the trigger to indicate which data needs to be read out; only data deemed useful is transferred off-chip and the rest is discarded [14,15]. Programmable regions of interest have been implemented in several ROICs [16,17,18] to read out select portions of the ROIC, reducing the amount of data being sent off-chip. In applications that require motion detection [19] in a larger stationary scene, applying real-time temporal filtering and sending out a reference frame followed by only the difference between subsequent frames could effectively reduce the total data being sent off-chip [20,21]. Compressive sensing techniques represent an additional method of data reduction as employed in [22,23], based on the principle that a small group of linear non-adaptive projections of a compressed image contains enough information for reconstruction. Several application specific schemes for data reduction are widely employed [24].

Several ROICs can be passively programmed to operate in different operating modes, such as photon-counting or photon time of arrival [25]. Some ROICs can simultaneously perform photon counting and time of arrival in different regions of the ROIC [26]. There are also several ROICs which offer a programmable number of bits in the data packet [27], and programmable exposure windows or time frames [28]. However, most of these modes are static operating modes, defined for either the entire ROIC or sub-sections of the ROIC at the beginning of an experiment, and are not dynamically controlled by the DAQ.

For real-time data processing, the DAQ needs to accurately identify the start and end of a frame to ensure data integrity. This is generally accomplished by sending a reset signal to the ROIC, which then transmits a well-defined frame header followed by data starting from the first pixel for a fixed well-defined frame in imaging. In zero-suppressed readout, since there is no predefined number of pixels transmitting data, the frame’s data structure changes constantly. Additionally, the time frame period can range from a few hundred nanoseconds to a few milliseconds. Whenever time frames, data packet length, or readout modes are changed dynamically, there is no seamless transition: the DAQ needs to stop and resynchronize, resulting in deadtime in the data stream. Hence, auto-synchronization techniques between the ROIC and DAQ are required to be able to create dynamically reconfigurable detector systems.

## 3. Concept of a Reconfigurable Detector System

**Figure 1:**
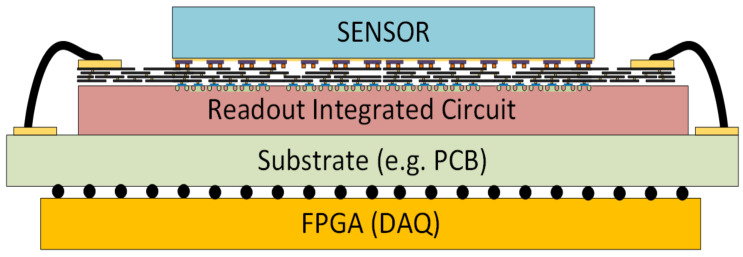
Detector system consisting of a sensor bonded to a ROIC and communicating with a data acquisition system such as an FPGA.

A specific example of a reconfigurable detector systems with adaptive - autonomous control over both temporal and spatial dimensions is presented. The sensor can dynamically receive feedback from the DAQ to select the optimum operating and readout mode. The implementation allows both advanced imaging techniques as well as data reduction at source. This mode change should not result in data loss or corruption in the ROIC or loss of synchronization in the DAQ, but should also not require excessively long headers to identify the start of a new frame. The concept of a reconfigurable detector system is shown in Figure 1.

**Figure 2:**
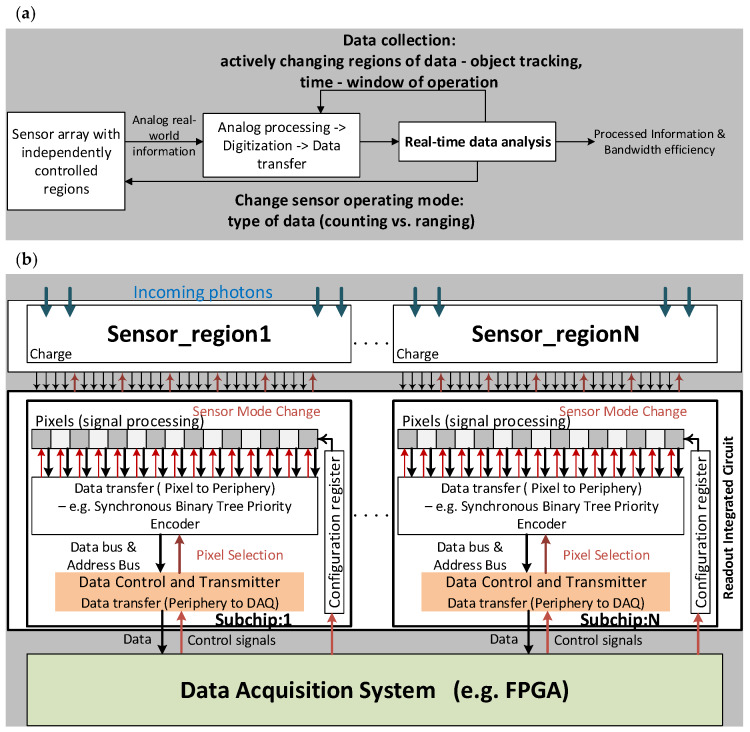
(**a**) Conceptual view of data flow. (**b**) System consisting of a sensor bonded to a ROIC with several independent, individually programmable subchips. Each subchip contains a peripheral data controller and transmitter (DCT). The DCT performs three critical functions, firstly it controls a synchronized binary tree priority encoder to transfer data from pixel to periphery, secondly it controls the output serializer to send data off-chip to an FPGA and thirdly it is also capable of being dynamically reconfigured by the FPGA in real-time. Data transfer is indicated with black arrows and control signals with red arrows.

A hybrid pixel detector composed of a sensor bonded to a ROIC, communicating with a Field Programmable Gate Array (FPGA) allows intelligent control of data generation. The data flows in both directions as shown in Figure 2a,b.The signal generated in the sensor is amplified and filtered by the analog front-end and digitized by the digital back-end of a pixel, in the case of a pixelated detector The digital data is transferred from pixel to periphery, for example by using a synchronized binary tree priority encoder (SB-PE) [29], which is managed by the data control and transmitter (DCT). The DCT also coordinates the transfer of data off chip to the FPGA. The FPGA can reconfigure the ROIC by changing the mode and time frames which defines the data being sent off-chip. Using a pixel configuration register it can further define regions of interest and other pixel level functions. Additional functionality in the ROIC can also change the sensor operating mode. With suitable sensors, reconfigurable ROICs can enable functions such as foveation, ensuring the requisite high speed and high resolution while efficiently reducing the amount of data produced at source. The ROIC can be divided into an array of independent sub-chips, each of which can be independently programmed [30] with its own independent DCT. For each sub-chip, the DAQ is capable of changing time frame (window of exposure), data packet length (number of data bits), as well as ROIC readout and sensor operating mode.

**Figure 3:**
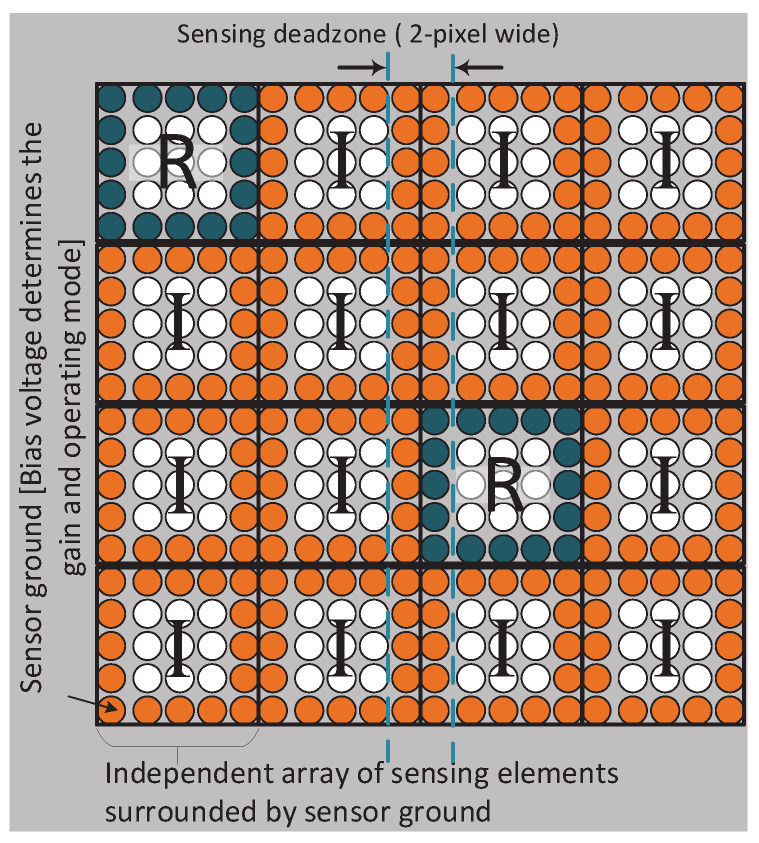
Concept of a reconfigurable sensor with an array of independently controlled segments, where each segment has an array of sensing pixels surrounded by a sensor ground. Changing the sensor bias voltage changes the internal gain of the sensor. Regions with a lower internal gain (orange) can be used as an imager (photon counting) and those with high gain (blue) can be used as a ranger (photon time of arrival). In this format the ground contacts lead to minimal dead zones or gaps between sensing areas, which can be designed to be <1% of the active area.

Figure 3 shows the concept of a reconfigurable sensor, with a few regions operating in a dynamic ranging mode with high internal gain and all others operating in a passive imaging mode with a lower internal gain (see details in the next section). The corresponding subchips of the ROIC would be operating in the photon time of arrival and photon counting mode respectively. As an example, one can imagine a large area camera system of 10 × 10 cm^2^ or larger, assembled by integrating a wafer scale sensor connected to an array of reticule size ROICs, each with multiple, independently, programmable regions (subchips) of approximately 2 × 2 mm^2^ [31]. These regions can be dynamically controlled by the DAQ based on the real-time analysis of the incoming data. For example, while observing a stationary scene, the entire sensor could be operating as a passive imager. Based on changes in the scene, the DAQ could reconfigure a well-positioned subchip to use its sensor and ROIC to compute the range to the emerging target of interest. Additionally, the time frame of nearby subchips could be reduced from milliseconds to a few hundred nanoseconds, and their mode changed from imaging to zero-suppressed, to observe the area near the target of interest at higher temporal resolution. This would allow the system to quickly track the target if it moves or expands. Furthermore, this technique reduces the amount of uninteresting data being sent off-chip by only sending out data about the target, rather than data about the entire scene.

### 3.1. Programmable Operating Modes for an Electron-Injection Sensor

Electron injector (EI) sensors are a class of heterojunction phototransistor detectors that combines a large-area optically absorbing layer with a nano-scale electronic active area [32,33]. By leveraging this additional degree of freedom in its geometry compared to conventional designs, this device architecture can simultaneously achieve high internal gain, high quantum efficiency, low noise and low dark current levels. As an example, InGaAs/InP-based EI devices with 1 µm injector diameter have been demonstrated, showing an optical gain in excess of 1000, room-temperature dark current of ∼1 nA and rise-time of ∼10 ns at 100 nW optical power. EI sensors have also been implemented into focal plane arrays (FPA), surpassing the performance of currently available commercial short-wavelength infrared (SWIR) cameras [34,35,36].

In its typical low-voltage operating conditions, the amplification mechanism of EI detectors is based on transistor action: namely the modulation of a potential barrier by trapped photo-generated excess carriers [37,38]. However, it has been observed that when the EI detectors are biased to higher voltage, a high enough field is created at the heterojunction to trigger Geiger-like operation [39,40]. In this operating condition, the amplification is based on avalanche processes, and the detector can be operated in Geiger mode [41].

**Figure 4:**
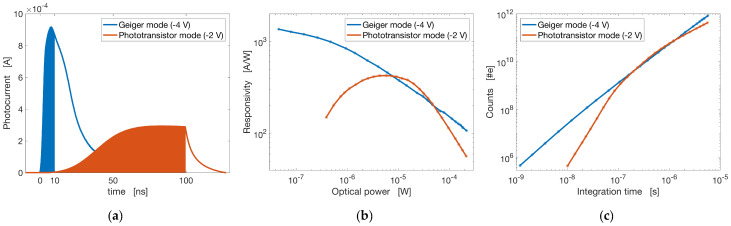
Photo-response of a 20 µm EI detector based on the architecture reported in [28], at 1 µW illumination power, in Geiger mode and phototransistor mode operation, corresponding for this device to applied bias of –4 V and –2 V respectively. (**a**) Time-resolved photo-response in the two operating modes: the shaded areas represent the charge accumulated on the detector during integration times $T_{int}$ of 10 ns and 100 ns for Geiger and phototransistor mode respectively. (**b**) Responsivity $R_{I}$ of the same device, as a function of optical illumination power, in the two operating regimes. (**c**) Electron count $N_{e}$ as a function of read-out integration time, obtained from the data in (**b**) by considering a constant illumination $\Phi_{0}$: so that $N_{e}=R_{I}\Phi_{0}T_{int}$.

Typically, as shown in Figure 4, Geiger mode operation enables a much faster response and higher gain at low photon flux, such that a shorter photon pulse can be resolved in this mode (for instance for ranging). As a result, Geiger mode operation allows obtaining a large signal from a shorter integration time compared to the phototransistor operation mode, thanks to the faster detector response and its higher gain. As clearly shown in Figure 4a,c, only Geiger mode operation is compatible with integration times of a few ns. This peculiar feature of EI detectors can be exploited to enable dynamic image foveation, by changing the detector bias using reconfigurable readout. Programmable operating modes can change the operating mode of EI sensors, and use them both for imaging and for ranging in independent regions of the camera. For instance, a staring array of EI detector could be operated at lower bias in the phototransistor regime, to take advantage of its lower power consumption and detection noise. Upon triggering by an event of interest, part of the FPA pixels could be switched in real time to operate in Geiger mode, enabling greater time-resolution imaging of the event of interest.

Finally, recent theoretical and experimental evidence have suggested that shrinking the injector size can further increase the EI detectors sensitivity, with the potential of improving both the speed and room temperature sensitivity of these devices. As considerable research effort is being devoted to advancing the detectors performance in this direction, the benefits of readout reconfigurability are expected to become even more significant. A photon-counting nanoscale EI detector array could be operated in Geiger mode for ranging, by determining the photon time of arrival, and in phototransistor mode for imaging, by counting the number of incoming photons.

### 3.2. Programmable Readout Modes In Roic

Based on the incoming photon count rate, the DAQ would be able to dynamically select and change the optimal time frame period, readout mode and the number of bits per pixel to ensure deadtime-less operation, with minimal data loss and optimal bandwidth usage. The reconfigurability of the ROIC is controlled by the data control and transmission block (DCT). The DCT in conjunction with the pixel configuration register can be externally programmed by the DAQ system to enable the ROIC to be either operated in synchronization, sparsification or full-frame imaging modes. These modes have different data packet lengths, and the number of data bits per pixel can be defined by the DAQ or user to be optimized depending on the length of the programmable time frame and the pixel occupancy requirements of the experiment.

#### 3.2.1. Synchronization Mode

**Figure 5:**
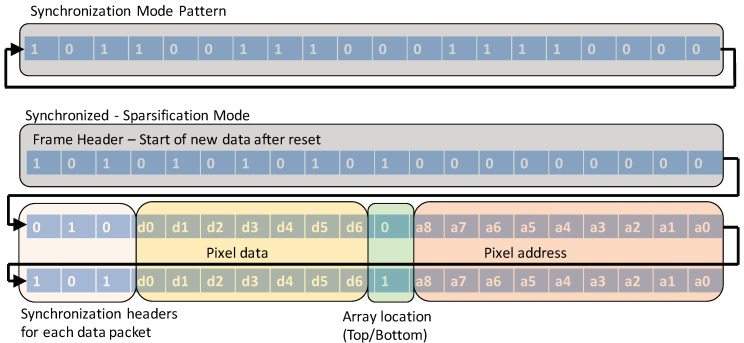
Synchronization pattern, frame header at the start of new data after DAQ reset and data packets with synchronization headers.

This mode is exclusively used to synchronize with the data acquisition (DAQ) system. A known pattern of data is continuously transmitted by the ROIC as shown in Figure 5, and the DAQ uses this mode to identify the start and end of a data packet as well as to maintain lock with the ROIC. Once the system is locked, the readout mode can be seamlessly changed to other modes. A synchronized-sparsification mode is also available which adds a short header to every data packet. This allows the DAQ to identify not just the start of a frame but also the start of a data packet. Anytime a DAQ looses lock, it could trigger this mode to resynchronize.

#### 3.2.2. Sparsification Mode

In the zero-suppressed format, all pixels with zeroes or no valid data are not read out. Since the total number and positions of pixels being read out are not known ahead of time, the system cannot rely on the transmission order to determine pixel position. Thus, this mode requires a packet based readout, where the data packet contains the address of the pixel along with the pixel data. As an example, a pixel array consisting of one serial output for a matrix of 1024 pixels would need a 10-bit unique pixel address. To minimize the address overhead several techniques involving a hybrid of packet based and frame based approaches have been implemented, and their efficiencies studied [42].

In the sparsification mode, irrespective of the readout speed, bandwidth is efficiently utilized when the pixel occupancy is below 50%, assuming 50% of the data packet is used by the pixel address. If over 50% of the pixels have data, it is more efficient to use the imaging mode to read out all pixels and thus avoid wasting bandwidth on addresses. However, for 2 bits of data per pixel and 10 bit address, 83% of the data packet is the pixel address, in which case the imaging mode with full frame becomes more efficient for pixel occupancies greater than 17%. It can be shown that for any given number of pixels i, a unique pixel address requires j bits, where:

(1) $$i\leq 2^{j}$$

**Figure 6:**
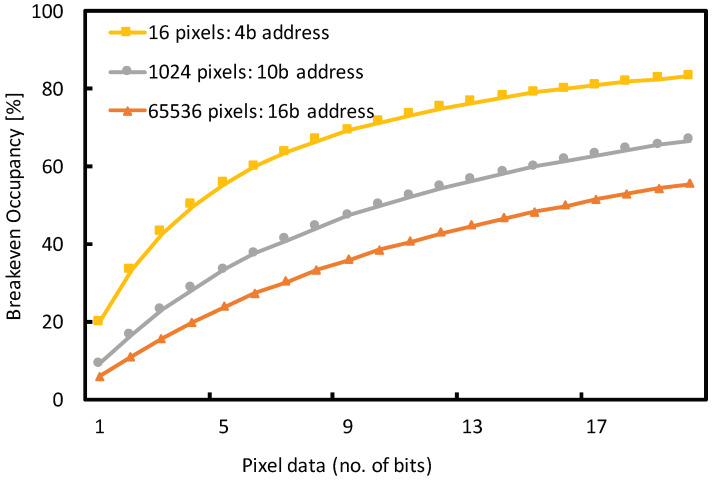
Break-even occupancy vs. number of pixel data bits per packet for pixel array sizes of 16, 1024 and 65,536 with 4, 10 and 16 address bits. When pixel occupancy is greater than the break-even occupancy, it is more efficient to read out all pixels rather than use zero suppression. The break-even occupancy of 50% is achieved when half of the data packet corresponds to the pixel address and the other half is pixel data.

If the data packet consists of j bits of address and k bits of pixel data, then regardless of readout speeds of a single serial output port, the break-even occupancy can be defined as:

(2) $$n=\frac{k}{j+k}\%$$

where it is equally efficient to use zero-suppression or full frame imaging. If the pixel occupancy is greater than n%, a frame-based readout is more efficient by reading out all pixels rather than using packet based zero-suppressed readout. Figure 6 shows the break-even occupancy for arrays with 16, 1024 and 65,536 pixels with 4-bit, 10-bit and 16-bit addresses respectively, for various pixel data length ranging from 1 to 20 bits.

The transmission of a single data packet with 10-bit address and 10-bit pixel data at 400 Mbps needs 50 ns. Similarly, reading out 10-bit address for pixels which had hits is 25 ns, providing only hit/no hit information. When reading out data within short time frames, the size of the data packet determines the data losses in a system. Hence, reducing the data packet length to match the time frame length is not only beneficial for bandwidth efficiency but can also reduce data losses in frames with more than the average occupancy of the application with incoming photon rate governed by Poissonian statistics.

Based on the pixel occupancy defined by the application, for a given address length and programmable number of data bits per pixel, the DAQ can reconfigure the ROIC to minimize data losses and/or maximize efficiency of the system.

#### 3.2.3. Imaging Mode

In full-frame imaging mode, every pixel in the matrix is read out in a predefined order, independently of the data register contents, so the pixel address does not need to be transmitted. In a photon-counting ROIC, for deadtime-less operation the total readout time must be less than the time frame or photon counting window. The frame rate itself is directly proportional to the output transmission speed and the number of parallel output ports and inversely proportional to the number of pixels and data packet length.

(3) $$\left(\frac{1}{\mathrm{frame}\;\mathrm{rate}}=\frac{\mathrm{no}.\;\mathrm{of}\;\mathrm{data}\;\mathrm{bits}\;\mathrm{per}\;\mathrm{pixel}\times \mathrm{no}.\;\mathrm{of}\;\mathrm{pixels}}{f_{serializerClk}\times \mathrm{no}.\;\mathrm{of}\;\mathrm{parallel}\;\mathrm{outputs}}\right)\leq \mathrm{Time}\;\mathrm{frame}\leq \frac{1}{\mathrm{Incoming}\;\mathrm{photon}\;\mathrm{rate}}$$

The optimum counter length which records the number of data bits can be chosen based on the rate at which the analog front-end can distinguish between two consecutive photons and the length of the time frame. For example, the shortest time frame in full-frame imaging mode with a data transmitting speed of 500 Mbps for 7 bit pixel data and one output for every 1024 pixels, is approximately 14 µs, with a frame rate of 70 Kfps. If the analog front-end can distinguish between two incoming photons (T_acq_) 120 ns apart, then in 15 µs, 128 photons/pixel can be recorded before the counter overflows. This defines the maximum sustainable photon count rate of approximately 40 G photons/(s cm^2^) for a pixel size of about 2500 µm^2^.

(4) $$\mathrm{Count}\;\mathrm{rate}\;\max=\frac{2^{\mathrm{no}.\;\mathrm{of}\;\mathrm{counter}\;\mathrm{bits}}}{\mathrm{pixel}\;\mathrm{area}*T_{acq}}$$

For applications in which specific predefined regions of interest smaller than the subchip size are required, a sub-frame based approach without the pixel address can be used for readout. A configuration register within each pixel contains a “set” bit and a “kill” bit, which indicates that irrespective of its contents, the pixel either needs to be read out or be ignored, respectively. Regions of interest can be defined by setting only a subset of pixels for readout while killing the rest of the pixels. Since only pixels that indicate that they have data are read out, and the readout order is always based on a position dependent priority, it would be easy to associate the data with the correct pixel. Moreover, this technique can be used to simultaneously define multiple regions of interest within a subchip.

Hence, by detecting the incoming photon flux, the DAQ would be able to dynamically select and change the optimal time frame period, readout mode and the number of bits per pixel to ensure both deadtime-less operation as well as efficient bandwidth usage.

## 4. Roic Reconfigurability Implementation, Results And Discussion

The data control and transmission (DCT) block controls the transfer of data from pixel to periphery, which in this case is performed by a synchronized binary tree priority encoder (SB-PE) [29] using a shared data bus and then serially transmits data off-chip through the output serializer.

It can be programmed using a 3-bit register to change readout modes and data packet lengths. In conjunction with the pixel configuration register, it can change the ROICs operating modes as well as define regions of interest. To ensure that time frame or mode changes do not result in data corruption, the DCT waits until the start of a new data packet to implement the change, allowing for a seamless transition. It can either generate and send a minimal known data synchronization pattern equal to two data packet lengths or repeat the last data again as the frame header. Since the address of a pixel can never be repeated twice, it is a unique pattern to identify the start of a frame. It is also capable of sending out a synchronization header with each data packet, if required. It generates a ‘done’ signal when all data has been transmitted off-chip and defaults to transmitting a known data pattern until the start of a new frame or it could be gated to stop transmission.

### 4.1. Mode and Data Packet Changes

#### 4.1.1. Implementation

**Figure 7:**
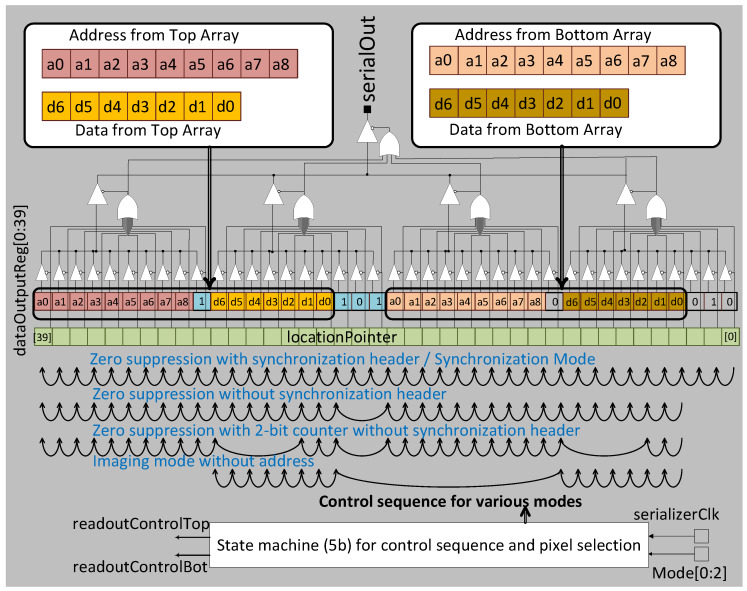
The output serializer consists of a 40-bit *dataOutput* register where the two top and bottom banks have their own independent 20-bit output registers The 40-bit *locationPointer* register with a single ‘1’ in its bit stream enables the tristate buffer allowing the corresponding *dataOutput* register bit to be sent off-chip. The *locationPointer* sequence is shown at the bottom for different operating modes.

To test reconfigurability and auto synchronization with the DAQ system, a test structure of the data control and transmission (DCT) block has been implemented in a 65 nm LP CMOS process. The full output data packet consists of 3-bit synchronization header, 7-bit pixel data and 10-bit pixel address. In this implementation the DCT alternately communicates with the top and bottom half of the pixel array through the SB-PE. The control signal *readOutControlTop/Bot* enables and disables a pixel in the top and bottom half of the array respectively. This interleaving is essential to allow sufficient time for data to settle once the pixel has access to the shared data transfer bus. The positive edge of *readOutControl* disables a pixel’s access to the data bus and the negative edge enables access for the pixel with the next highest priority. The *dataOutputReg* in the DCT uses a 40-bit register, where the two halves of the array have their own independent 20-bit output registers. A 3-bit globally programmable mode in conjunction with a 5-bit state machine sets a 40-bit *locationPointer* register in parallel to the 40-bit *dataOutputReg* register to determine the output data packet length and readout format as shown in Figure 7.

At any given time, only 1 bit of the *locationPointer* register is ‘1’ and the rest of the bits are ‘0’. Depending on the user programmable mode, the ‘1’ cycles through either all the registers or only a few pre-selected registers. Depending on the pattern of bits selected, the corresponding *dataOutput* register bit is enabled for readout. Upon reset, the location pointer starts at [0] and transmits a known data pattern defining the start of a frame, before it defaults to the chosen mode of operation.

The output stage of the DCT uses a two-stage tristate buffer for transmitting the output data as shown in Figure 7. The location pointer enables the *dataOutputReg* content to be transferred off-chip. To reduce glitches and parasitics on the *serialOut* pin, this is accomplished by a two stage OR gate. Ten outputs are ORed together to create an intermediate output step and then subsequently the four outputs are ORed to enable the correct *serialOut*.

#### 4.1.2. Results

**Figure 8:**
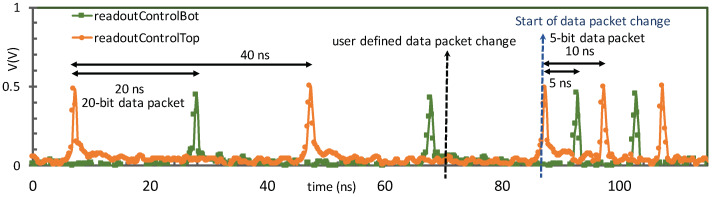
The DCT is operating at 1 GHz and a user defined data packet change to reduce the pixel data occurs in the middle of data transmission sequence. The output serializer finishes sending the current data packet completely before changing the data packet length from 20 bits to 5 bits.

To analyze the speed constraints of the DCT, the test structure was characterized using an externally programmable shift register in lieu of receiving data from an array of pixels. The control speed of the DCT partly determines the full-frame imaging rate as well as the shortest time frames in zero-suppressed mode. The readout speed also relies on data transfer from pixel to periphery as well as output line drivers. The state machine in the DCT can be programmed to change the data packet length. Figure 8 shows the output serializer transitioning from transmitting a 20-bit packet in zero-suppressed mode to 5 bits per data packet in full-frame imaging mode. The DCT ensures that regardless of when the mode change is applied to the ROIC, the existing data a sent off-chip before the change is allowed to take effect within the ROIC. The DCT is capable of generating control signals at 1 GHz.

#### 4.1.3. Discussion

The interleaving of data provides adequate time for the pixel data to settle before it is latched by the DCT. However, in a zero-suppressed readout mode if a majority of pixels with data are located only in one half of the pixel array, then this scheme forces the other half of the array to send data packets with “0s” off-chip, thereby reducing the bandwidth efficiency. To circumvent a position-dependent readout inefficiency, instead of dividing the matrix into top-bottom or right-left halves, two halves can be created by choosing alternate pixels in a chequerboard pattern. This would however increase the data transfer bus capacitance as the total area occupied by the two halves would increase by a factor of two. The subdivision of the array into two halves allows lower bus capacitance and higher operating speeds, and further segmenting into smaller array sizes is feasible at the expense of larger area for the serializer. In 65 nm process the DCT occupies 70 × 10 µm^2^, hence additional registers, is not a major concern.

Although the DCT can operate at 1 GHz, the data transfer from the pixel to the periphery is the speed bottleneck: a maximum error free data transmission rate of 550 Mbps was measured for the worst case, when all the bits of the shared data bus are forced to transition from 1 -> 0 -> 1 for consecutive data readout cycles [29]. Higher transfer rates could be achieved by using lower voltage signaling [43,44].

### 4.2. Frame Changes and Autosynchronization with The Daq

#### 4.2.1. Implementation

The synchronized binary tree priority encoder (SB-PE) [29] operates in conjunction with the DCT as an arbitration tree. Within any given time frame, each pixel with valid data sends a request signal to the DCT for read out through the priority encoder. The SB-PE establishes a position-dependent order in which the pixels are allowed to transfer data to the output serializer. By interleaving data between two halves of the pixel array, the output serializer transmits data from periphery to off-chip for one half of the array while simultaneously transferring data of the other half from the pixel to the periphery. The time frame or the window of exposure is defined by the *frameClk* period. The rising edge of the *frameClk* is used by the priority encoder to create a new priority list by assigning the order in which multiple pixels need to be read out.

**Figure 9:**
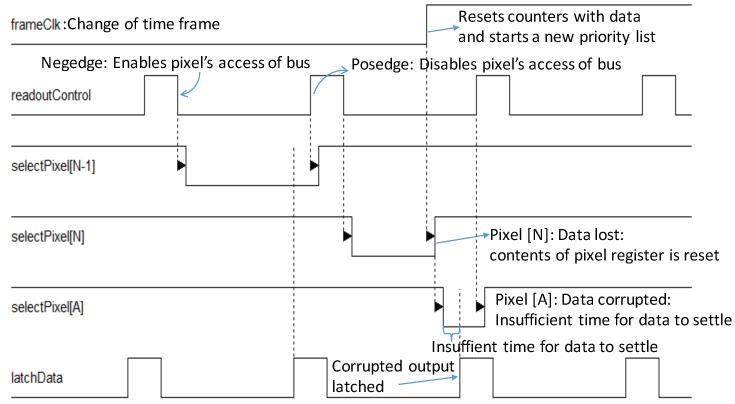
At *frameClk* change the contents of the pixel register currently being readout are reset, resulting in data loss. The priority encoder subsequently enables the first pixel from a new priority list. However, there is insufficient time for the data to settle, again resulting in corrupted data. The change of *frameClk* therefore requires a mechanism to ensure no data loss or corruption.

The rate of data transfer is defined by the *serializerClk* with a time period of few nanoseconds, whereas the frame readout period, defined by the user programmable *frameClk*, is from a few hundreds of nanoseconds to a few milliseconds. These two clocks controlling the readout of the ROIC are independent and not synchronized. Unlike the *serializerClk*, which is physically localized in the DCT within a few hundred µm^2^, the *frameClk* is distributed across the pixel matrix over a few mm^2^. It is unnecessary and inefficient to synchronize these two independent clock signals. The rising edge of the *frameClk*, triggers a frame change in every pixel. This triggers the data registers participating in the ‘readout’ to reset and those that are ‘recording’ data are changed to ‘readout’. Those that are ‘idle’ do not change. The pixel that is currently transferring data is the most affected by this change: the data that it is transferring gets reset, causing corrupted data to be latched at the periphery. Simultaneously, a new priority list is created, and a new pixel gets access to the data bus, but if there is insufficient time for the new data to settle and transfer to the periphery, the highest priority data is also lost, as shown in Figure 9.

**Figure 10:**
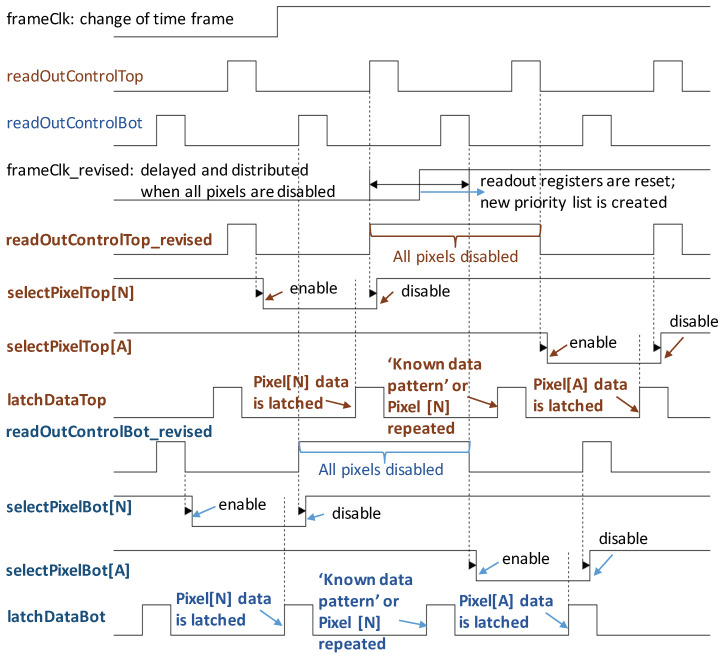
Modified control sequence to maintain data integrity at frame changes. When dynamically changing time frames, data loss and corruption are avoided by disabling all pixels while the *frameClk* is propagated across the ROIC. While the pixels are disabled a known data pattern or the previous pixel data is transmitted off-chip to identify the start of a frame.

To ensure no data loss or corruption and to maintain synchronization with the DAQ, every time the time frame is changed at the positive edge of *frameClk*, the transmission of the current data packet must be completed, before the *frameClk* is propagated across the ROIC to every pixel. Moreover, the pixel array must be disabled while the *frameClk* is distributed across the ROIC. Since the negative edge of the *readOutControl* signal enables the pixel, this edge is delayed modifying the pulse width. In lieu of the data from the pixel, either a known header is transmitted or the last data sent off-chip is repeated. In full frame imaging, where no address is read out, a known header with length of two data packets is used for synchronization. In sparsification mode, since no two pixels can have the same address, repeating the data twice indicates the start of a new frame to the DAQ. Meanwhile the rising edge of the *frameClk* across the chip creates a new priority, and finally at the negative edge of the *readOutControl* signal, the pixel with the highest priority is given access to the data bus, as shown in Figure 10. This change in *frameClk* is seamless, without either distorting the data being sent off-chip or losing the new data generated by a change in the priority list. This is an improvement to the scheme presented in [45], since the last pixel data of the previous frame are not lost.

#### 4.2.2. Results

**Figure 11:**
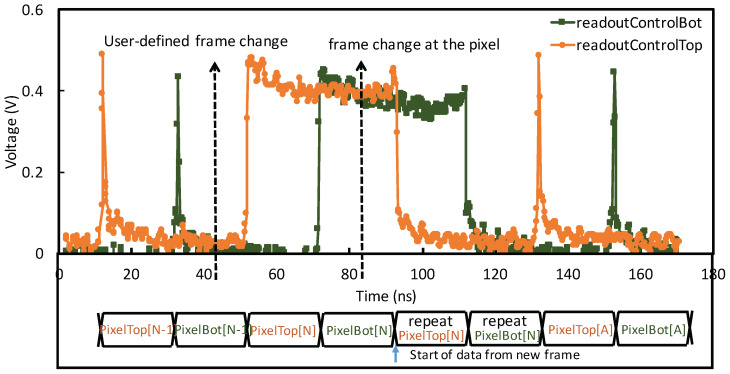
Frame clock change for the DCT operating at 1 GHz. A frame change at the positive edge of *frameClk* is issued at approximately 40 ns, initiating the new frame at approximately 90 ns, with the DCT operating at 1 Gbps.

Figure 11 shows the measurement results for the DCT test structure. A time frame change caused by the posedge of *frameClk* occurs at 40 ns, the DCT disables all the pixels in the array by delaying the *readOutControlTop/Bot* negedge. It then allows the *frameClk* to propagate across the pixel array at 80 ns and allows sufficient time to compensate for skews and delays. Upon reaching the pixel, the posedge of *frameClk* resets the counters from the previous frame and a new priority list is established. The negedge of *readOutControl* subsequently enables the pixel with the highest priority. The output data stream shows the repeated data indicating the frame change.

#### 4.2.3. Discussion

The DAQ monitors a few data packets immediately after initiating the frame change to identify the start of the new time frame. Depending on the implementation, either locating the known data pattern or repeated data packets would indicate the start of the new frame. If a periodic resynchronization is desirable, for example to cope with high rates of single event upsets in high-radiation environment, the ROIC could be configured in the synchronized sparsification mode to identify data packets. The monitoring of the address data, which for pixels in the same frame should always be in decreasing order due to the position-based priority, could also be exploited by the DAQ to flag synchronization errors.

## 5. Conclusions

A technique for implementing a dynamic control of the readout mode of a ROIC without loss of synchronization and with minimal overhead was presented. This technique is especially suited for dynamically changing readout and operating modes in a camera system. With the application of this scheme, the time associated with processing and interpreting data by the DAQ likely becomes the dominant latency contributor in implementing real-time dynamic readout mode change.

As a concrete example of application of the presented method, a reconfigurable camera system is designed with an independently biased, segmented electron injection sensor bonded to a reconfigurable ROIC with matching independent, segmented subchips, and controlled by an FPGA in real-time. A large scale system with multiple sensors, ROICs and FPGAs can be envisioned. A reconfigurable ROIC that utilizes the high-speed data control and transmitter working in conjunction with the low power, synchronized, binary tree priority encoder can be dynamically controlled by the data acquisition system to enable a new class of reconfigurable detector systems. These systems will be capable of having multiple foveation centers in a large area detector system while efficiently utilizing data bandwidths and can become platforms for implementing real time machine-learning algorithms. Reconfigurable functions include frame based, region-of-interest based and zero-suppressed packet-based readouts with programmable number of pixel data bits. The time frame can also be programmed from hundreds of nanoseconds to tens of milliseconds based on incoming photon flux. A method has been implemented which allows seamless dynamic changes of time frames without loss or corruption of data. The data control and transmitter has several synchronization features such as well-defined short frame headers, unique data patterns at frame changes as well as synchronization headers within data packets to maintain lock with the DAQ.

Although this reconfigurable approach has been presented for camera systems, the same techniques can be applied to IoT devices as well as distributed sensors used in various industrial applications.

## 6. Patents

Fahim, Farah, and Grzegorz W. Deptuch. “Edgeless large area ASIC.” U.S. Patent 10,352,991, issued 16 July 2019.
